# American Society of Dental Surgeons

**Published:** 1850-10

**Authors:** 


*American Society of Dental Surgeons.*—The last meeting of this Association, held the 13th, 14th and 15th of August, at Saratoga, was very well attended, although several of the members who intended to have been present, were prevented from doing so. The President of the Society, Dr. E. Parmly, and the Recording Secretary, Dr. A. Westcott, to the regret of all present, were both absent; the former, in consequence of an injury of one of his ancles, which confined him to his house; and the latter, in consequence of the sickness of one of his children. Two other members, who, as we were informed, had gone to New York, with a view of proceeding to Saratoga to attend the meeting, were prevented from doing so, in consequence of having mistaken the time when it was to take place. They were under the impression that it had been appointed for the first instead of the second Tuesday in August, and not being able to remain from home so long, were compelled to return before the meeting took place. The mistake occurred in this way: nearly all the former regular meetings had been appointed for the first Tuesday in August; but when the called meeting of March last adjourned, it did so, through inadvertence, until the second Tuesday, and the notices of the Recording Secretary were issued accordingly; but several of the members, failing to receive the notice, supposed the meeting would take place at the usual time, the first Tuesday in August.

It will be perceived from the minutes of the meeting, which we publish in another part of the Journal, that the resolutions passed at a former meeting, several years ago, requiring each member to sign a pledge not to use amalgam, have been rescinded. The reasons which determined the Society to do this, are so clearly set forth in the report of the commiitee to whom the matter was referred at the called meeting held in March last, as to render any comments or explanation from us unnecessary. We may state, however, in a word, that they were based upon the belief that the resolutions had accomplished the object for which they were designed, and that there no longer existed any necessity for their enforcement.

For a detailed account of the proceedings of the Society, the reader is referred to the minutes.

